# Colloidal Silicon Dioxide in Tablet form (Carbowhite) Efficacy in Patients with Acute Diarrhea: Results of Randomized, Double-Blind, Placebo-Controlled, Multi-Center Study

**DOI:** 10.1038/s41598-020-62386-0

**Published:** 2020-04-14

**Authors:** Vadim Tieroshyn, Larisa Moroz, Oleksandra Prishliak, Lyudmila Shostakovich-Koretska, Oksana Kruglova, Lyudmila Gordienko

**Affiliations:** 1 Kharkiv National Medical University, Kharkiv, Ukraine; 2 Vinnytsya National Medical University named after M. I. Pirogov, Vinnytsya, Ukraine; 3 Ivano-Frankivsk National Medical University, Ivano-Frankivsk, Ukraine; 4 Dnipro State Medical Academy, Dnipro, Ukraine; 5 Lugansk State Medical University, Rubizhne, Ukraine; 6 Kiev City Clinical Hospital No.9, Kiev, Ukraine

**Keywords:** Clinical trial design, Drug development, Drug development, Clinical trial design, Clinical trial design

## Abstract

The acute diarrhea is a wide-spread disease. The prescription of enterosorbents is appropriate as a primary measure for the treatment of the acute diarrhea for effective prevention of the fluid and electrolyte loss, as well as method for symptom relief of the attack of the disease. Aim of the study - the antidiarrheal efficacy and safety study of high-dispersion silicon dioxide enterosorbent in tablet dosage form in patients with acute diarrhea. This was randomized, double-blind, placebo-controlled, 4-center study. Acute diarrhea was defined as three and more episodes of watery stool per day either during 48 hours or less before study entry in the patients having normal stool recently. It has been postulated that symptoms and signs of acute diarrhea have to be caused by direct infection of the gastrointestinal tract and did not associated with moderate-to-severe systemic states. 144 patients with established acute diarrhea were randomized into treatment group (enterosorbent “Carbowhite”, n = 120) or placebo group. Date collection including severity diarrhea, systemic symptoms was performed at baseline and daily during 7 days. Stool examination and serological assay were performed at baseline. The primary end points were declared as time to complete recovery from acute diarrhea. It has been found that the use of the siliceous enterosorbent (“Carbowhite”) allowed to reduce (p < 0.001) the treatment period averagely for 0.9 days (95% confidence interval 0.5–1.2 days) in comparison with placebo. Data of safety monitoring has revealed that both patient groups had negative stool culture, while initiation of antibiotic treatment was run more frequently in placebo group (8.3%) compared to investigational product group (4.1%, P = 0.044). The siliceous enterosorbent “Carbowhite” was well tolerated and reduced the recovery time of the acute episode of the diarrhea in the clinically significant form.

## Introduction

The acute infectious diarrhea is a challenge for the contemporary medicine, although the leading factors contributing in the pathogenesis of the disease is well known^1^. However, there is a large body of evidence regarding higher rate of morbidity and mortality in developing countries due to diarrheal diseases^2,3^. Moreover, the acute diarrheal diseases are also wide-spread in the countries with high sanitary-hygienic level^3^. Foodborne Disease Burden Epidemiology Reference Group finds that the global burden of diarrheal diseases is comparable to those of the major infectious diseases, as HIV/AIDS, malaria and tuberculosis^4^.

The attacks of diarrhea are usually brief and self-limiting, but the symptoms cause discomfort and are incapacitating^4^. It is well-known that these symptoms lead to the substantial social marginal costs, as it is supposed that the half of the occurred episodes results in working days lost^5^. The acute diarrhea is also the most frequent health disorder in travellers. Traveller’s diarrhea leads to the substantial discomfort as a result of the interference in the travelling routes, changes of business opportunities and revenue from tourism^6^. Prevention of traveler’s diarrhea should be ideally based on the dietary restrictions, but the clinical experience demonstrates that this aim is extremely difficult to achieve^7,8^.

Symptomatic therapy is still the important aspect of acute diarrhea treatment^9^. There are several options of acute diarrhea treatment, including oral rehydration, anticholinergic, antisecretory and antibacterial medicinal products, probiotics and intestinal adsorbents^10–15^. Intestinal adsorbents are a group of specific products, which adsorb and excrete present in the intestine gases, pathogens, allergens, exotoxins and endotoxins from the body. Based on safety and efficacy data of enterosorbents administration, it has been concluded that administration of these products can be recommended as a primary tool for the acute diarrhea treatment for rapid and effective prevention of fluid and electrolyte loss and symptom improvement^16,17^. The aim of the study was to investigate the antidiarrheal efficacy and safety of the siliceous enterosorbent (“Carbowhite”) in original tablet form in patients with acute diarrhea.

## Methods

The initial hypothesis that is tested in the study is whether the treatment with original enterosorbent “Carbowhite” is better than placebo to reduce recovery period in patients with acute diarrhea.

### Study design

It was a randomized, double-blind, placebo-controlled, 4 centres’ study. The study was registered in ClinicalTrials.gov (Identifier: NCT03633344, first posted 16/08/2018, result first posted 06/06/2019).

### Sample size calculation

The sample size calculation was made using G*Power 3.1.5 (Heinrich-Heine-Universität Düsseldorf, 1992–2013) (alpha error = 0.05, beta error = 0.80). Total number of selected participants was calculated as 165 patients including drop-out percent (1%)^18^. A final number of randomised individuals was 144 (120 for active treatment and 24 for placebo in ratio 5-to-1) to achieve estimated treatment effect for the placebo and silicon dioxide group equal 1 day and more. An anticipated percent expected treatment effects for the placebo and treatment groups were defined as completed recovery time of acute diarrhea and this period has been expected for 2 days and 1 day after treatment run-in, respectively. Based on this assumption we have finally calculated sample size to identify the difference in primary effect between placebo and investigating product equal 1 day and more. The confidence level is defined as 95%, confidence interval was indicated as 1 day, and estimated sample size was 124 patients.

### Patients’ population

**Figure 1:**
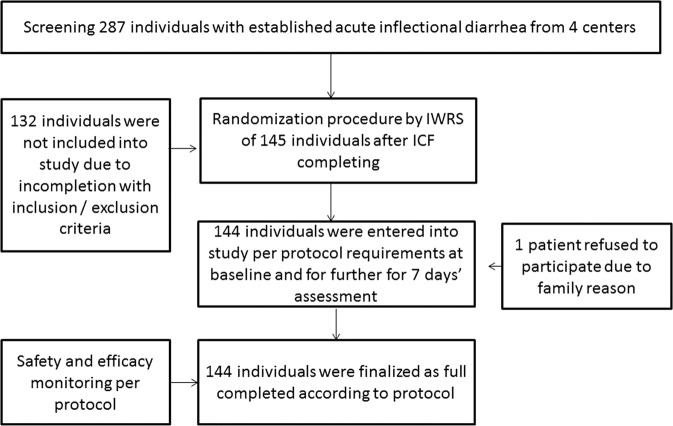
Flowchart for screening and randomization procedures.

Figure 1 is presented a flow chart with screening, randomization, treatment and monitoring procedures across the study. The patients were recruited from an inpatient hospital ward, office visit, and emergency room. Patients’ inclusion criteria were male and female subjects, age from 18 to 55 years old; acute diarrhea (more than 3 episodes of watery stool a day) during 48 hours or less in patients with normal stool usually; body temperature ≤38 °С; patient’s ability to adequately cooperate during the study, and written informed consent. Patients’ exclusion criteria were age <18 or >55 years old; the blood or pus in stool; the body temperature >38 °С; the episodes of acute diarrhea for the last 30 days; food intolerance-related diarrhea; medication related diarrhea including antibiotic-associated diarrhea, functional diarrhea; systemic bacterial infections; travel-associated systemic infections including malaria, dengue fever and SARS; HIV infection associated diarrhea; *Clostridium difficile* infection; other extra intestinal infections; the administration of anti-diarrheal medicinal products during 24 hours; salmonellosis (*Salmonella* of any serotype, different from *S. typhi* and *S. paratyphi*; ICD-10 code: A02.0), dysentery (*Shigella dysenteriae*, *Shigella flexneri, Shigella boydii*; ICD-10 code: A03.0-A03.3), escherichiosis (*Escherichia coli*; ICD-10 code: A04.0-A04.4) requiring prescription of antibacterial drugs; pregnancy, lactation; concomitant decompensated diseases or acute conditions, in investigator’s opinion, are able to effect on the study results; alcoholism and drug abuse; participation in any other clinical studies.

### Acute diarrhea determination

Conventionally, acute diarrhea without relation reason (infection or non-infection) is defined as an abnormally frequent discharge of semisolid or fluid fecal matter from the bowel, lasting less than 14 days. Acute infectious diarrhea was defined as three and more episodes of watery stool per day during 48 hours or less before study entry in patients with initially normal stool^19^. It has been postulated that symptoms and signs of acute diarrhea have to be caused by direct infection of the gastrointestinal tract and did not associated with moderate-to-severe systemic states^20^. Any extra intestinal reasons leading to acute diarrhea, as well as non-infectious causes related to inflammatory bowel disease, were not considered ethological factors for acute diarrhea^20,21^. Patients with other non-infectious diarrhea that affected extra intestinal organ systems, such as food intolerance, medication related diarrhea, functional diarrhea, were not enrolled as candidates for the study participants.

### Randomization procedure

Previously 1:1 randomization was planned to minimize restricted other randomizations, which can be associated with small groups, and prevent from statistical bias due to incorrect validation of disease severity in different study centres^22,23^. However, Steering Committee of Health Ministry of Ukraine has proposed to randomly allocate all enrolled patients in 5:1 ration (treatment versus placebo group) to minimize a placebo effect. According to the inclusion/exclusion criteria 144 patients from four centres were enrolled in this study. Patients were randomized into treatment and placebo groups with IWRS (Interactive Web Response System) system^22^. Participants were randomly assigned following simple randomization procedures (computerized random numbers) to investigational product (IP) group or placebo^24^. Finally, the 1^st^ group (120 patients) administered the IP siliceous enterosorbent (“Carbowhite”), the 2^nd^ group (24 patients) administered placebo.

### Treatment regimen

Every tablet of “Carbowhite” contains 210 mg of high-dispersion silicon dioxide with the area of active sorption surface no less than 300 square meters per one gram of a substance, and was produced by the licenced producer Pharmex Group LLC (Ukraine). Every tablet of placebo was produced identically to the investigational enterosorbent without active ingredients (with powder of chalk) by the same producer.

The siliceous enterosorbent (“Carbowhite”) or placebo was prescribed to the patients with acute diarrhea in the following dosage regimen: the investigational enterosorbent/placebo were prescribed in the dose: 3 tablets per one administration (210 mg × 3 = 630 mg) four times a day (630 mg × 4 = 2520 mg). At insufficient anti-diarrheal efficacy (no reduction of stool frequency (for a day) from the moment of beginning of a patient’s treatment), it was possible to increase the dose in two steps: step 1 − 4 tablets per one administration (210 mg × 4 = 840 mg) four times a day (840 mg × 4 = 3360 mg) during 1–2 days, and step 2–5 tablets per one administration (210 mg × 5 = 1050 mg) four times a day (1050 mg × 4 = 4200 mg) for Day 2, Day 3, Day 4 of treatment. Study duration for each patient was 7 days.

#### End point determination

The primary endpoint was run as a time to complete recovery from acute diarrhea.

#### Data collection

Research coordinators collected data daily, completed a standardized, and refined electronic case report form^25^. Complaints, clinical status, anamnesis, medical and family history, concomitant medication, adverse events, and new medications were collected at screening, at baseline and at every visit of the patients in the hospital. It has been planned a call of patients to investigator if needed.

All patients were previously screened systemic infections, including HIV, hepatitis, tuberculosis, as well as inflammatory bowel disease and autoimmune disease. Clostridium difficile Infection was excluded using contemporary procedure^26^. Cultural and serological investigations were performed according to contemporary protocol to rule-out salmonellosis, dysentery, and escherichiosis as it was described above^27^.

All contacts were specified in source documents and verified by principal investigator. Stool frequency was reported by the patient using personal original diary, with was reviewed by investigator before observation at the visit. The diary was validated for this study and approved by Institutional Review Board before study entry. Stool consistency was assay using validated Bliss Stool Classification System^28^, which has been completed at the visit and then reported. All completed charts were collected and stored as source document. All collected data were reported on-line through completing electronic case report forms. Additionally we reported all endpoints as soon as they had appeared and reported them to the monitor(s) and collected data around these events. Reporting of the events included identification of adverse events or not, specification of incidence, severity and timing or duration of them.

#### Blinding procedure

The study was constructed to be blinded participants being enrolled, included and treated, clinical staff administering, and the team interpreting results. However, data collectors were independent and they were unaware of the treatment the participant received^29^. In fact, all participants and study staff were blinded and the study was defined as double blind study.

#### Unblinding procedure

The unblinding process was embedded onto study protocol and the participant and/or study team of which treatment the participant received during the study had a possibility to disclose the investigating product (active drug or placebo) using IWRS^30^. To protect participants in the event of medical or safety reasons unblinding procedure could be made due to request of central advisor committee, IRB, staff person, or authority if needed. After unblinding procedure patient was excluded from the study and it did not run to participate in the study again^31^.

#### Efficacy monitoring

The primary efficacy parameter was a percentage of patients administered the investigational product (IP) who has gained the efficacy endpoint (the reduction of defecation to 3 times a day, no liquid stool). The secondary efficacy parameter was a percent of patients administered the IP who has discontinued the study, the average treatment duration and average quantity of tablets for treatment course. The end treatment criteria were reduction of stool frequency to 3 and less times a day, bacteriological diagnosis verification with the consequent antibacterial therapy beginning, development of adverse reactions associated, in the opinion of the physician-investigator, with the administration of the IP/placebo. During the study the concomitant therapy with other sorbents, probiotics, prokinetics and antimicrobial medicines was prohibited.

#### Safety monitoring

The primary safety parameter was a percentage of patients administered the IP who had undesirable effects (adverse reactions/adverse effects – AR/AE). The secondary safety parameter was dynamics of vital sign change, dynamics of body weight change, and laboratory test values. Data regarding a number of patients received positive and negative stool culture, as well as frequency of antibiotics initiating were reported.

#### Anthropometry

Anthropometric measurements including body weight and height were made at the baseline using standard procedures.

#### Declaration on ethical principles

This study was performed in accordance with the Law of Ukraine “On medicines” (No. 124/96-BP, in the wording of 19.06.2016), the Declaration of Helsinki and the Procedure for Conducting Clinical Trials of Medicinal Products and Expert Evaluation of Materials Pertinent to Clinical Trials and Model Regulations on the Ethics Committee. The study has been performed upon authorization and under control of the State Expert Centre of the Ministry of Health of Ukraine and finished on Oct 7, 2016 (National Identification number was CRICOD-7-180).

Local Ethical Committees of Vinnytsya City clinical hospital No. 1, Ivano-Frankivsk regional infectious diseases clinical hospital, Kyiv City clinical hospital No. 9, Dnipro City clinical hospital No. 21 approved the study protocol in Febr 10, 2016, Febr 22, 2016, Febr 10, 2016 and Febr 18, 2016 respectively.

All individuals included in the study have given written informed consent. The informed consent forms (ICFs) were approved by an appropriate regulatory chair of the State Expert Center of the Ministry of Health (Ukraine) and were updated as needed.

#### Statistics

We used MedCalc v.16.8.4 (MedCalc Software Inc., Broekstraat, Belgium), G*Power 3.1.5 (Universität Kiel, Germany) for statistics^32^. Quantitative variables were expressed as mean (M) and standard deviation (±SD), median and interquartile range (IQR), estimated marginal mean (95% confidence interval [CI]) or number (percentage). An independent group t-test was used to compare all the interval parameters matching the criteria of normality and homogeneity of variance. For interval parameters that fail to match these criteria, the non-parametric Mann-Whitney test was used to compare variables. The Fisher’s Exact Test and Chi-square Test were used at comparison of efficacy of treatment vs. placebo effect. During analysis of the dynamics of parameter changes, ANOVA methods for the repeated measurements (or their non-parametric analogue) were used. The logistic regression models were used for the potential risk factors for treatment failure. The ROC (receive operation curve characteristics) analysis was used to evaluate the logistic regression model. The power of the model’s predicted values to discriminate between positive and negative cases is quantified by the Area under the ROC curve (AUC). Odds ratio (OR) index and its 95% CI were calculated for assessment of the extent of the factors effect within the frameworks of the logistic regression model. The risk ratio (RR) indexes and the average number of patients who need to be treated (Number-Needed-to-Treat NNT) and their 95% CI were used for assessment of the IP efficacy in comparison with placebo. Odds ratio (OR) index and its 95% CI were calculated for assessment of the extent of the factors effect within the frameworks of the logistic regression model. P value between groups has been chosen equal to 0.05.

## Results

The study population has consisted of 144 subjects (85 males and 59 females) with mean age 31.5 years (from 18 years to 58 years). The baseline data of eligible individuals are listed in Table 1. The statistically significant difference in distribution by gender between subgroups has not been detected (p = 0.45). Consequently, there was no statistically significant difference in parameter value in both subgroups at the baseline (p > 0.05).

**Table 1:** The patient characteristics at the baseline.

Parameter	Mean value			P value
	Placebo subgroup (n = 24)	Treatment subgroup (n = 120)	Entire group (n = 144)	
Age, Me (IQR), years	34.8 (23.9–47.6)	30.9 (22.9–35.0)	31.5 (23.3–36.1)	0.20
Male/female, n	12/12	73/47		
${\text{Height},}^{\overline{{X}}}$ ±SD, cm	173.1 ± 8.1	175.4 ± 8.6	175.0 ± 8.5	0.23
Weight, Me (IQR), kg	70.5 (65.0–84.5)	72.5 (66.5–80.5)	72.0 (65.5–81.0)	0.99
Diastolic BP, Me (IQR), mm Hg	80 (70–80)	80 (70–80)	80 (70–80)	0.84
Systolic BP, Me (IQR), mm Hg	120 (110–120)	120 (112.5–120)	120 (110–120)	0.50
HR, Me (IQR), b/min	83.0 ± 5.4	81.7 ± 6.2	81.9 ± 6.0	0.31
T,${}^{\overline{X}}$ ±SD, °С	37.55 ± 0.28	37.44 ± 0.51	37.46 ± 0.31	0.54
${\rm{Stool}}\,{\rm{frequency}}{,}^{\overline{X}}$ ±SD, n	7.8 ± 2.1	7.0 ± 2.4	7.1 ± 2.3	0.12
Dose, tablets	12	12	12	1.0

Abbreviations: Me − median; IQR − 27%-75% interquartile range; BP − blood pressure; SD − standard deviation; HR − heart rate; Т − body temperature.

24 patients administering placebo and 120 patients administering the IP (“Carbowhite”) have been treated during the first 24 hours. The dynamics of the analysed parameters is represented in Table 2. The analysis of treatment efficacy among the patients administered with IP/placebo have yielded that sufficient clinical improvement (i.e., 3 defecation acts, no watery stool) has achieved in 59 patients in the IP group and in 1 patient only in the placebo group at the end of the first treatment day.

**Table 2:** The dynamics of parameter changes on the 1st day of treatment.

Parameter	Mean value		P value
	Placebo group (n = 24)	Treatment group (n = 120)	
Weight, Me (IQR), kg	70.5 (65.0–84.5)	73.0 (66.5–80.5)	0.98
DBP, Me (IQR), mm Hg	77.5 (70–80)	80 (70–80)	0.45
SBP, Me (IQR), mm Hg	120 (117.5–120)	120 (117.5–120)*	0.24
${\rm{HR}}{,}^{\overline{X}}$ ±SD, b/min	74.9 ± 3.3*	74.7 ± 3.8*	0.79
T,${}^{\overline{X}}$ ±SD, °С	36.78 ± 0.30*	36.70 ± 0.22*	0.26
${\rm{Acts}}{,}^{\overline{X}}$ ±SD, times	5.0 ± 1.9*	3.7 ± 2.3*	<0.001
${\rm{Dose}}{,}^{\overline{X}}$ ±SD, tablets	13.2 ± 1.9	12.4 ± 1.2	—

Abbreviations: IQR – interquartile range; T – body temperature; Me – median; SBP – systolic blood pressure; DBP – diastolic blood pressure; HR – heart rate; SD – standard deviation.

The parameter values after 2^nd^, 3^rd^, 4^th^ days of treatment are represented in Table 3. After two days of the treatment, the effect has achieved in other 36 patients in the IP group, the total number of patients who have successfully finished the active treatment period with the IP was 95 individuals. Eleven patients in the placebo group have finished treatment.

**Table 3:** The dynamics of parameter changes during the treatment.

Parameter	Mean value 2^nd^ day of treatment			Mean value 3^rd^ day of treatment			Mean value 4^th^ day of treatment		
	Placebo group (n = 24)	Treatment group (n = 120)	P value	Placebo group (n = 24)	Treatment group (n = 120)	P value	Placebo group (n = 24)	Treatment group (n = 120)	P value
Weight, Me (IQR), kg	71.0 (65.5–84.8)	73.0 (67.0–80.0)	0.95	76.5 (67.5–86.5)	73.0 (65.0–78.0)	0.10	78.0 (63.5–99.0)	71.5 (68.0–73.0)	0.47
DBP, Me (IQR), mm Hg	75.0 (70–80)	80 (70–80)	0.86	77.5 (70–80)	75 (70–80)	0.86	75.5 (70–79)	70 (70–75)	0.32
SBP, Me (IQR), mm Hg	120 (120–120)	120 (120–120)	0.20	120 (120–120)	120 (120–120)	0.10	120 (120–120)	120 (120–120)	0.56
HR, Me (IQR), b/min	74.1 ± 2.7	73.9 ± 2.6	0.49	74.1 ± 2.8	73.1 ± 3.9	0.36	74.0 ± 2.5	74.0 ± 3.6	>0.99
$T,{}^{\overline{{X}}}$ ±SD, °С	36.64 ± 0.15	36.63 ± 0.21*	0.43	36.62 ± 0.10	36.60 ± 0.15	0.39	36.57 ± 0.08	36.58 ± 0.10	0.63
${\rm{Acts}},{}^{\overline{X}}$ ±SD, times	3.5 ± 1.9*	2.8 ± 1.4*	0.10	2.8 ± 1.1*	2.0 ± 0.9*	0.03	2.1 ± 0.9*	1.3 ± 0.5*	0.09
${\rm{Dose}},{}^{\overline{X}}$ ±SD, tablets	13.3 ± 1.9	12.2 ± 1.0	—	13.7 ± 2.2	12.0	—	12.0	—	—

Abbreviations: IQR – interquartile range; T – body temperature; Me – median; SBP – systolic blood pressure; DBP – diastolic blood pressure; HR – heart rate; SD – standard deviation.

In three days of treatment (Table 3), clinical efficacy has been observed in other 23 patients in the IP group (total number of successfully treated patients is 118) and among 8 patients in the placebo group (total number of successfully treated patients is 20).

**Figure 2:**
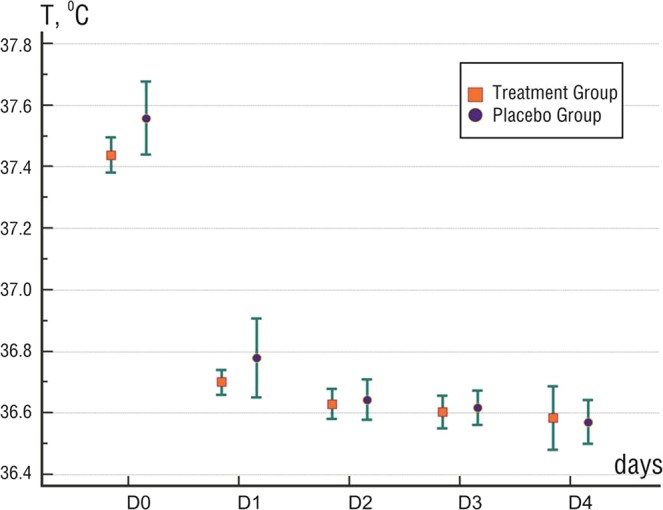
The dynamics of body temperature changes in patients. Note: Mean value and 95% CI are shown.

**Figure 3:**
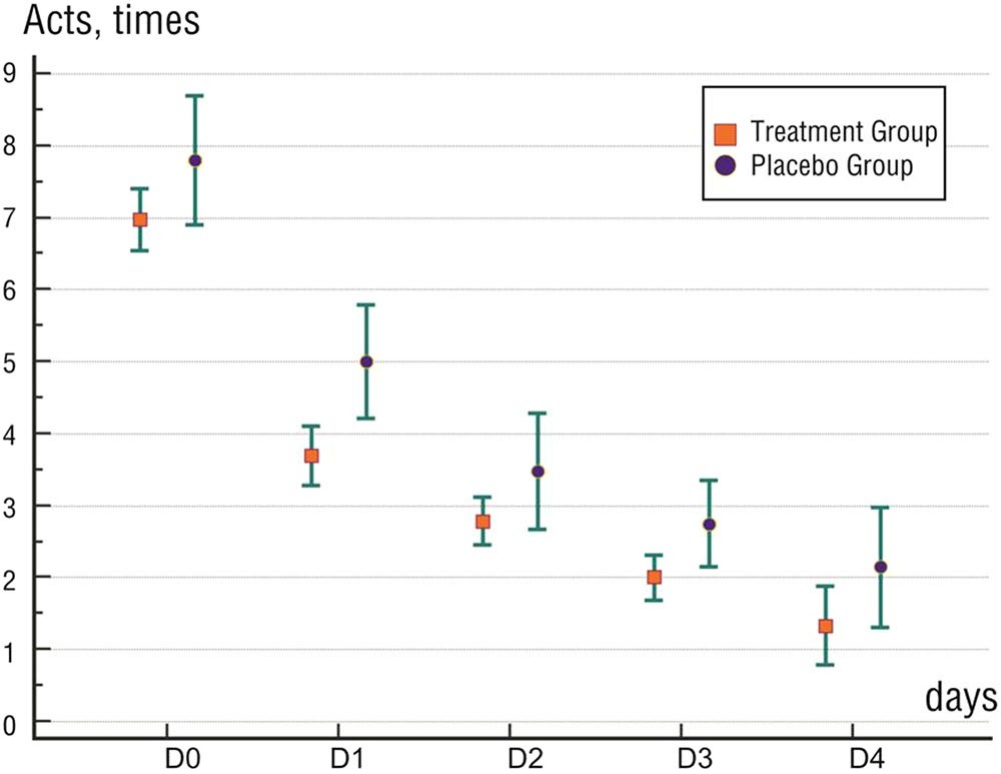
The dynamics of acts in patients. Note: Mean value and 95% CI are shown.

On the 4 day of the observation period (Table 3), the treatment with the IP/placebo has finished for the rest of 2 patients in the IP group, and 4 patients in the placebo group. All patients in both the IP and placebo groups have completely recovery at the end of four day. The dynamics of body temperature and acts in randomized patients are represented in Figs. 2 and 3.

### The analysis of treatment failure risk

**Figure 4:**
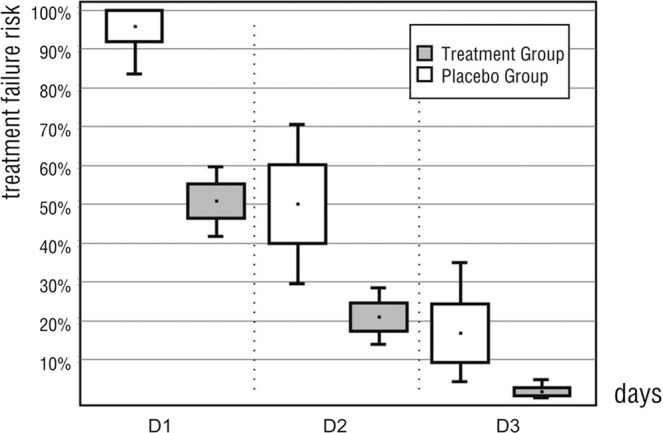
The dynamics of treatment failure risk changes. Note: Risk level, its standard error and 95% CI are shown.

Patient treatment results of 2 groups are represented in Table 4. Analysis has revealed that on the 1^st^, 2^nd^ and 3^rd^ day the treatment failure risk in the IP group was lower (p < 0.05 for all cases) than in the placebo group (Fig. 4). Consequently, on the 2^nd^ day of the treatment (control point) the treatment failure risk in the placebo group was 50% (95% CI 29.5–70.5%), and in the IP group it was 20.8% (95% CI 14.0–28.6%). It was associated with a 2.5-fold reduction of the risk (p = 0.01) in the IP group (RR = 0.4 (95% CI 0.3–0.7) (NNT = 3.4 (95% CI 2.0–11.2)).

**Table 4:** The dynamics of treatment efficacy changes.

Effect		Abs. (M ± m%)		P value	RR (95% CI)
		Placebo group (n = 24)	IP group (n = 120)		
1^st^ day	Not achieved	23 (95.8 ± 4.1)	61 (50.8 ± 4.6)	<0.001	0.5 (0.4–0.6)
	Achieved	1 (4.2 ± 4.1)	59 (49.2 ± 4.6)		
2^nd^ day	Not achieved	12 (50.0 ± 10.2)	25 (20.8 ± 3.7)	0.01	0.4 (0.3–0.7)
	Achieved	12 (50.0 ± 10.2)	95 (79.2 ± 3.7)		
3^rd^ day	Not achieved	4 (16.7 ± 7.6)	2 (1.7 ± 1.1)	0.03	0.10 (0.02–0.52)
	Achieved	20 (83.3 ± 7.6)	118 (98.3 ± 1.1)		
4^th^ day	Not achieved	—	—	—	—
	Achieved	24 (100)	120 (100)		

Abbreviations: M – mean; m – standard error of the mean; RR – risk ratio; CI – confidence interval; Abs. – absolute value.

### The univariate and multivariate logistic regression analysis

The univariate logistic regression analysis has revealed significant reduction (p = 0.004) of treatment failure risk at the use of the IP (OR = 0.26 (95% CI 0.11–0.66) versus placebo (Table 5). The multivariate logistic regression model of the IP effect on the treatment failure risk was performed after adjustment to the patient’s age, sex, body weight index, severity index at baseline (quantity of stool acts), and temperature at baseline. The multivariate logistic regression model was adequate (χ^2^ = 21.5 at one degree of freedom, p = 0.002). The increase in the treatment failure risk for increased severity index at baseline for each act was determined (OR = 1.24; 95% CI 1.04–1.47 p = 0.015).

**Table 5:** The parameter values of univariate and multivariate logistic regression model of forecasting the treatment failure risk.

Factor	Model parameter value, b ± SD	P value	OR (95% CI)
**Univariate logistic regression model**			
Constant	0.00 ± 0.41	—	—
Treatment, investigational enterosorbent vs placebo	−1.36 ± 0.47	0.004	0.26 (0.11–0.66)
**Multivariate logistic regression model**			
Constant	0.00 ± 0.41	—	—
Treatment, investigational enterosorbent *vs* placebo	−1.00 ± 0.50	0.047	0.37 (0.13–0.98)
Frequency of stool	0.21 ± 0.09	0.015	1.24 (1.04–1.47)
Age	0.031 ± 0.022	0.171	—
Body Mass Index	0.00 ± 0.07	0.998	—
Male *vs* Female	−0.80 ± 0.45	0.075	—
T	0.75 ± 0.73	0.302	—

Abbreviations: b – beta coefficient; OR – odds ratio; CI – confidence interval; T – body temperature; SD – standard deviation.

### The analysis of investigation product number

The performed analysis demonstrates that in the IP group the average number of tablets for 3 acts was 12 pcs. (Q_I_ = 12 pcs. – Q_III_ = 24 pcs.), minimum/maximum quantity of tablets was 12 pcs and 48 pcs, respectively. In the placebo group the average number of tablets for 3 acts was 30 pcs. (Q_I_ = 24 pcs. – Q_III_ = 36 pcs.), minimum/maximum quantity of tablets was 12 pcs and 48 pcs, respectively.

### The analysis of end point for the study

The primary end points have been declared as time to complete recovery from acute diarrhea. The analysis has demonstrated that the mean value of the treatment duration to the achievement of full clinical effect in the placebo group was 2.6 days (95% CI 2.3–3.0 days) and in the IP group it was 1.7 days (95% CI 1.6–1.9 days). Consequently, the use of the IP allowed significant (p < 0.001) reducing the treatment duration averagely for 0.9 days (95% CI 0.5–1.2 days) in comparison with placebo.

During analysis, the primary efficacy parameter was used as output variable Y. For patients for whom efficacy endpoint was achieved on the 2^nd^ day and earlier, the value Y = 0 (107 patients), or alternatively Y = 1 (37 patients). Patient age, sex, and body weight, severity index at baseline (quantity of acts), temperature at baseline, and also the treatment procedure (IP/placebo) were used as the factors of further analysis.

**Figure 5:**
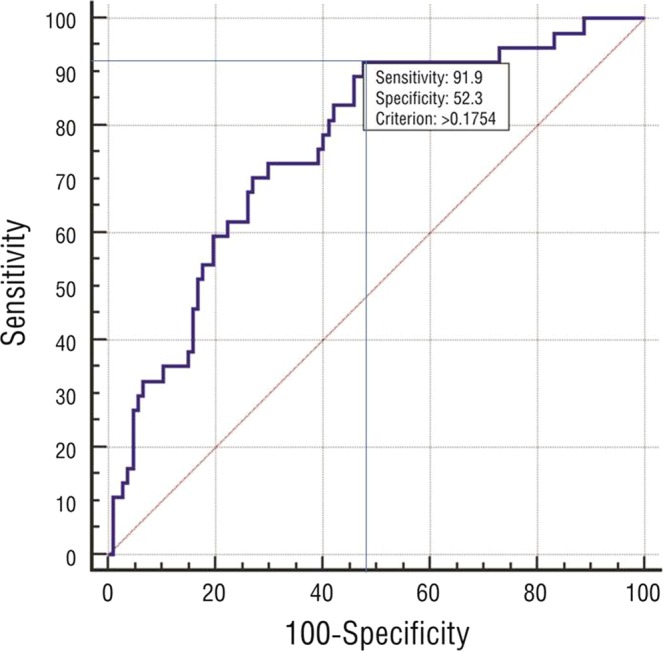
The prediction of the treatment failure risk; the results of the ROC curve analysis. Note: the best balanced cut-off point = 0.1754 was selected by maximization of Youden test.

Univariate logistic regression model was used to detect the effect of the IP to reduce recovery period in comparison with placebo. The obtained model was adequate (χ^2^ = 8.0 at one degree of freedom, p = 0.005). The received operating characteristic curve analysis has shown that AUC for placebo was 0.61 (95% CI 0.52–0.69) and for IP it was 0.76 (95% CI 0.68–0.83; p < 0.05). The best balanced cut-off point obtained by maximization of Youden test was 0.1754 (Fig. 5). The treatment failure risk adjusted by age, sex, body weight index, severity index (quantity of acts) and temperature in the IP group was lowered (OR = 0.37 (95% CI 0.13–0.99; p = 0.046) in comparison with placebo.

### Safety monitoring

Data of safety monitoring has revealed that both patient groups had negative stool culture, while initiation of antibiotic treatment was run more frequently in placebo group (8.3%) compared to IP group (4.1%, P = 0.044) (Table 6).

**Table 6:** Data of safety monitoring in and placebo groups during the study.

Variables	Placebo group (n = 24)		IP group (n = 120)		P value
	n	%	n	%	
Stool culture received (n)	24	100	120	100	1.0
Positive stool cultures (n)	0	0	0	0	1.0
Initiated antibiotics treatment (n)	2	8.3	5	4.1	0.044

Thus, IP treatment was associated well with positive response from clinical status, reducing recovery period and good safety of the therapy.

## Discussion

The data of the study have revealed that estimated efficacy of the tablet dosage form of the investigational enterosorbent (“Carbowhite”) in patients with acute diarrhea was superior placebo. The results of the study have yielded that IP treatment was significantly better when compared with placebo among patients with acute diarrhea having negative stool culture. Yet, a risk of treatment failure was found to be minimized in the IP group when compared with placebo.

Previous clinical observations have revealed that the most of the patients with acute diarrhea had a self-limiting disease course^33,34^, the main challenge for the physician is to discriminate patients for whom symptomatic therapy is sufficient from those with severe disease course and threatening complications^34,35^. There are several clinical guidelines with well-shaped methodology for prevention and treatment of acute diarrhea taken into consideration several factors that contribute in a prolongation of diarrhea persistence^34–36^. For instance, the antimotility drug loperamide and the secretion inhibitor racecadotril are considered as the first choice for the treatment of acute uncomplicated traveler’s diarrhea including traveler’s diarrhea and non-infection diarrhea^35^. To note, a large number of patients with acute diarrhea who generally demand antibiotic therapy should be disabused of their expectations, and antibiotics should use only in complicated cases. Several clinical conditions, in which bacterial agents were not confirmed in stool specimens and a risk of *Clostridium deficile* infection is low, were associated with hypovolemia, hemodynamic disorders and electrolyte imbalance due to the prolongation of diarrhea^37^. Moreover, antibiotic use is agents with high potency to prolong diarrhea due to deteriorative effect on the microbiota^36,37^. In this context, other therapeutic approaches including enterosorbents’ use are currently available for the treatment of acute diarrhea. Indeed, previous clinical studies and meta-analysis have shown that tannin albuminate in combination with ethacridine lactate have provided 36% protection from acute diarrhea, but both drugs were not widely available^38–40^. Among the non-antimicrobial agents that were available and useful for the treatment of the patients with acute diarrhea was a bismuth subsalicylate-containing preparation, which has demonstrated decrease in the passage of loose stools by 16%-18%. The anti-secretory and anti-motility agent loperamide reduced the passage of loose stools by approximately 50% and has been especially useful, in combination with antimicrobial agents, in reducing the total duration of post-treatment diarrhea to a matter of hours^38–41^.

Undoubtedly, greater attention should be paid to the monitoring and supervision of antibiotics as well as safety and tolerability of the any drugs that have been implemented into diarrhea treatment scheme. In fact, stool culture negative patients with acute diarrhea did not get benefit from acute or chronic antibiotic treatment, such as decrease in a need in hospital admission and hospital stay^39^. In this context, enterosorbent like “Carbowhite” may have promising clinical efficacy regarding serge of completed recovery in diarrhea.

Although mild-to-moderate acute diarrhea in patients with negative stool examination might have spontaneously recovery^40,41^, IP insured diminish of diarrhea symptoms. However, there are several difficulties to conduct a study of a disease where most patients seek no care and many spontaneously resolve quickly^42^. Having a risk of pseudomembranous colitis and microbiota disorders after antibiotic use, enterosorbents may be promising drugs to recover diarrhea, because they have excellent profile of safety and tolerability. Interestingly, placebo group gained >7 kg from the first day to fourth day that was probably associated with fluid retention after diarrhea recovery, while the implementation of the enterosorbent “Carbowhite” did not relate to this effect. There is evidence regarding oral intestinal enterosorbent Enterosgel (Polymethylsiloxane polyhdrate) that was orally administered materials which pass through the gut where they bind various substances^43^. Despite it is recommended as a symptomatic treatment for acute diarrhea and it also demonstrated interactions with bile acids and numerous pharmaceutical drug including antibiotics, there is not face-to-face comparison between Enterosgel and Carbowhite, as well as between Enterosgel and placebo^44^. Additionally, adsorption capacity for Enterosgel has varied, whereas this parameter for “Carbowhite” was dramatically lower.

However, large clinical trials are required to be provided to clearly explain the therapeutic profile of the enterosorbent “Carbowhite” in patients from several populations and various clinical conditions associated with acute diarrhea. Thus, based on interval estimation, the probability of the undesirable effects associated with the use of investigational enterosorbent is less than 1.6% (p = 0.05). Similar studies to study the efficacy of the enterosorbent “Carbowhite” had not previously been conducted.

Other clinical studies dedicated several methods of treatment of acute diarrhea have revealed numerous controversies^45,46^. For instance, antibiotics have resolved acute diarrhea within 7 days (median was 3 days) after initiation of treatment versus 17 days in the placebo group^45,46^. However, this effect was closely associated with broad spectrum of enteric protozoan pathogens and bacterial infections^47,48^. Our study has shown that the difference between placebo and IP in diarrhea recovery period was 0.9 day, but the patients enrolled in the study did not exhibit positive stool culture. Therefore, acute diarrhea related to travel requires to be treated with antibiotics regardless of severity of disease to complete recovery^49^.

The enterosorbent “Carbowhite” has been developed in Ukraine. It has a complex composition based on the features of high-dispersion colloidal silicon dioxide (SiO_2_). Silicon dioxide blocks the receptors of mucous membrane responsible for pathogens adhesion and toxins binding, thus, accelerating the adsorption of active substances in the intestine^17,50,51^. Moreover, silicon dioxide that is embedded in the structure of “Carbowhite” does not represent direct damage impact on intenstinal wall and it does not provoke electrolyte disturbances^52^. Importantly, there is no evidence regarding of accumulation of silicon dioxide in organs and tissues of animals was observed at its intake^17^. The drug does not contain sugar; it can be used in patients with diabetes^17^. Additionally, silicon dioxide promotes the movement from the internal environment of the body into the digestive tract of various toxic products, including medium molecules, oligopeptides, amines and other substances, due to concentration and osmotic gradients, followed by binding and excretion from the body^52,53^. Therefore, there is a large body of evidence that silicon dioxide-based enterosorbents might have pleiotropic effects beyond adsorption of the intestinal products. It is been suggested that silicon dioxide may block the adhesive factors and cause denaturation of the adhesive proteins on the bacterial cell surface, disturbing the functional activity and adhesive properties of the pathogenic microorganisms^16,17^. Antimicrobial activity of silicon dioxide against enteric pathogens defines its anti-diarrheal effect; three-dimensional structure of the sorbent increases viscosity of the liquid media, causing anti-diarrheal and coating effect^53^.

Thus, the randomized, double-blind, placebo-controlled, multi-center study efficacy of the administration of colloidal silicon dioxide in tablet form (“Сarbowhite”) in patients with acute diarrhea was revealed that the high-dispersion silicon dioxide enterosorbent (“Carbowhite”) exhibited much more antidiarrheal effect when compared with placebo. The treatment with the enterosorbent “Carbowhite” versus placebo is well tolerated by patients and does not cause adverse effects. These findings support the role of “Сarbowhite” in acute diarrhoea especially in vulnerable groups where rapid resolution of symptoms is required.

### Study limitations

The study design was limited by a small number of patients who were taking the investigational enterosorbent. The second limitation affects the method of randomization. Takin into consideration these previously reported findings we have decided that the 1:1 allocation during randomization was used in the study to prevent traditional bias regarding uncertain outcomes in patients with mild-to-moderate acute diarrhea that might recover spontaneously. We have allocated all patients’ number via IWRS through four centers in Ukraine, but Steering Committee of Health Ministry of Ukraine has not approved this randomization ratio and proposed changing from 1:1 to 5:1 aimed minimize a placebo effect in patients. We agree that this is a bias, but this limitation should be taken into consideration further. The next study limitation is various etiologies of diarrhea, while infections as primary cause of the diarrhea were rule out in the study. Investigations of the quality of life among patients with diarrhea treated with - the enterosorbent “Carbowhite” are recommended to carry out in the future.
